# Intravenous nicardipine for Japanese patients with acute intracerebral hemorrhage: an individual participant data analysis

**DOI:** 10.1038/s41440-022-01046-4

**Published:** 2022-10-13

**Authors:** Kazunori Toyoda, Sohei Yoshimura, Mayumi Fukuda-Doi, Adnan I. Qureshi, Manabu Inoue, Kaori Miwa, Masatoshi Koga, the ATACH Trial Investigators, Adnan I. Qureshi, Yuko Y. Palesch, Kazunori Toyoda, Kazuyuki Nagatsuka, Masatoshi Koga, Masafumi Ihara, Yongjun Wang, Nobuyuki Sakai, Takayuki Hara, Zhimin Wang, Jiann-Shing Jeng, Sachin Agarwal, Kiwon Lee, Stephan A. Mayer, M Fareed K. Suri, Qaisar A. Shah, Jawad F. Kirmani, Adnan I. Qureshi, Haitham Hussein, Jill M. Novitzke, Cathie Witzel, Bo Connelly, Saqib A. Chaudhry, Emily I. Abbott, Erik T. Maland, Kathryn A. France, Basit Rahim, Zachariah Miller, Alfredo J. Caceres, Logan J. Brau, Mushtaq H. Qureshi, Jessy K. Thomas, Mohammad R. Afzal, Norrita Rech, Yuko Y. Palesch, Renee Martin, Wenle Zhao, Lydia Foster, Jaime Speiser, Catherine Dillon, Jaemyung Kim, Cassidy Conner, Adam Henry, Kristina Hill, Kristen Clasen, Christy Cassarly, Daniel F. Hanley, Carlos S. Kase, J. Ricardo Carhuapoma, Nichol McBee, Claudia Moy, Scott Janis, J. Claude Hemphill, Brian L. Hoh, Mario Zucharello, Michael K. Parides, the SAMURAI Investigators, Kazuomi Kario, Michito Namekawa, Jyoji Nakagawara, Kenji Kamiyama, Eisuke Furui, Ryo Itabshi, Yukako Yazawa, Yoshiaki Shiokawa, Kazutoshi Nishiyama, Yasuhiro Hasegawa, Hisanao Akiyama, Satoshi Okuda, Tomoko Noda, Hioshi Yamagami, Kenichi Todo, Kazumi Kimura, Kensaku Shibazaki, Yoshiki Yagita, Yasushi Okada, Tomonaga Matsushita, Takanari Kitazono, Teruyuki Hirano, Shoji Arihiro, Shoichiro Sato, Masaki Naganuma, Koichiro Maeda, Mayumi Mori, Tomohisa Nezu, Tetsuya Miyagi, Kaoru Endo, Masato Osaki, Junpei Kobayashi, Takuya Okata, Yuki Sakamoto, Eijirou Tanaka, Haruka Kanai, Azusa Tokunaga, Kazuo Minematsu

**Affiliations:** 1 Department of Cerebrovascular Medicine, National Cerebral and Cardiovascular Center, Suita, Japan; 2 Center for Advancing Clinical and Translational Sciences, National Cerebral and Cardiovascular Center, Suita, Japan; 3 Zeenat Qureshi Stroke Institute and Department of Neurology, University of Missouri, Columbia, MO USA; 4 University of Minnesota, Minneapolis, MN USA; 5 Medical University of South Carolina, Charleston, SC USA; 6 National Cerebral and Cardiovascular Center, Suita, Japan; 7 Beijing Tiantan Hospital, Beijing, China; 8 Kobe City Medical Center General Hospital, Kobe, Japan; 9 Toranomon Hospital, Tokyo, Japan; 10 The First People’s Hospital of Taizhou, Taizhou, China; 11 National Taiwan University Hospital, Taipei, Taiwan; 12 Columbia University, New York City, NY USA; 13 St. Cloud Hospital, St. Cloud, MN USA; 14 Abington Memorial Hospital, Abington, PA USA; 15 New Jersey Neuroscience Institute, JFK Medical Center, Edison, NJ USA; 16 Zeenat Qureshi Stroke Research Center, University of Minnesota, Minneapolis, MN USA; 17 Data Coordination Unit, Department of Public Health Sciences, Medical University of South Carolina, Charleston, SC USA; 18 Division of Brain Injury Outcomes, Johns Hopkins University, Baltimore, MD USA; 19 National Institute of Neurological Disorders and Stroke of the National Institutes of Health, Bethesda, MD USA; 20 Jichi Medical University School of Medicine, Shimotsuke, Japan; 21 Nakamura Memorial Hospital, Sapporo, Japan; 22 Kohnan Hospital, Sendai, Japan; 23 Kyorin University School of Medicine, Mitaka, Japan; 24 St Marianna University School of Medicine, Kawasaki, Japan; 25 National Hospital Organization Nagoya Medical Center, Nagoya, Japan; 26 Kobe City General Hospital, Kobe, Japan; 27 Kawasaki Medical School, Kurashiki, Japan; 28 National Hospital Organization Kyushu Medical Center, Fukuoka, Japan; 29 Kyushu University, Fukuoka, Japan; 30 Kumamoto University, Kumamoto, Japan

**Keywords:** Acute stroke, Antihypertensive therapy, Blood pressure, Hypertension, Intracranial hemorrhage

## Abstract

The effects of acute systolic blood pressure levels achieved with continuous intravenous administration of nicardipine for Japanese patients with acute intracerebral hemorrhage on clinical outcomes were determined. A systematic review and individual participant data analysis of articles were performed based on prospective studies involving adults developing hyperacute intracerebral hemorrhage who were treated with intravenous nicardipine. Outcomes included death or disability at 90 days, defined as the modified Rankin Scale score of 4–6, and hematoma expansion, defined as an increase 6 mL or more from baseline to 24 h computed tomography. Of the total 499 Japanese patients (age 64.9 ± 11.8 years, 183 women, initial BP 203.5 ± 18.3/109.1 ± 17.2 mmHg) studied, death or disability occurred in 35.6%, and hematoma expansion occurred in 15.6%. Mean hourly systolic blood pressure during the initial 24 h was positively associated with death or disability (adjusted odds ratio 1.25, 95% confidence interval 1.03–1.52 per 10 mmHg) and hematoma expansion (1.49, 1.18–1.87). These odds ratios were relatively high as compared to the reported ones for overall global patients of this individual participant data analysis [1.12 (95% confidence interval 1.00–1.26) and 1.16 (1.02–1.32), respectively]. In conclusion, lower levels of systolic blood pressure by continuous intravenous nicardipine were associated with lower risks of hematoma expansion and 90-day death or disability in Japanese patients with hyperacute intracerebral hemorrhage. The impact of systolic blood pressure lowering on better outcome seemed to be stronger in Japanese patients than the global ones.

## Graphical abstract

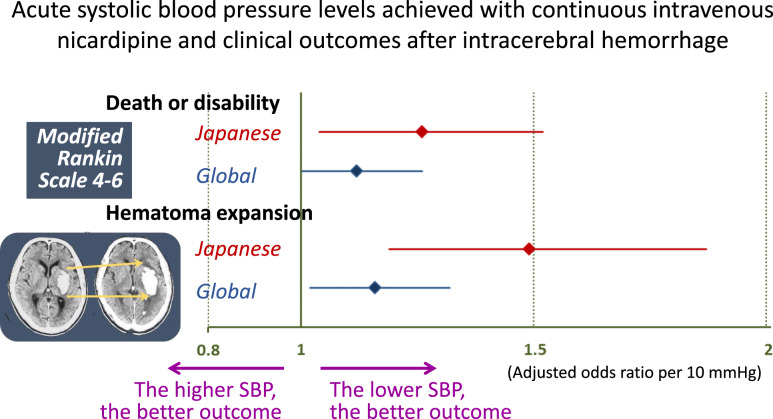

## Introduction

Acute lowering of elevated blood pressure (BP) is one of the few promising therapies for acute intracerebral hemorrhage (ICH) [1–3] that reportedly has relatively high incidence in Asia, including Japan [4]. Nicardipine, a dihydropyridine-derived calcium channel blocker is rapidly titratable, has a short half-life and few side effects, and produces peripheral arterial vasodilatation without affecting cardiac conduction pathways and, accordingly, has a low risk of bradydysrhythmias [5]. Thus, this agent is recommended as a first-line agent for acute cerebrovascular emergencies. We performed a meta-analysis using individual participant data (IPD) and showed that a lower level of mean hourly systolic BP (SBP) achieved during the initial 24 h by continuous intravenous administration of nicardipine was associated with lower risks of early hematoma expansion and 90-day death or disability in acute ICH [6]. In the study, SBP was especially strongly associated with the both risks in Asian participants, showing significant treatment-by-subgroup (Asians versus non-Asians) interactions.

Japanese participants accounted for 39% of overall participants in the meta-analysis. In the present study using the same IPD database, we determined the effects of acute SBP levels achieved with intravenous nicardipine for Japanese ICH patients on clinical outcomes.

## Methods

This meta-analysis using IPD is registered with PROSPERO (CRD42020213857). Details of the study design have been reported previously [6]. Briefly, PubMed was searched for relevant articles published before October 1, 2020 with the search terms regarding ICH, BP, and nicardipine. Prospectively-registered clinical trials or observational studies involving adults who started to receive intravenous injection of nicardipine within 12 h after onset of ICH, whose hourly SBP levels were available during the first 24 h, and whose functional outcome at 90 days or later was assessed using the modified Rankin Scale (mRS, ranging from 0 to 6 with higher scores indicating worse functional disability) with >20 patients enrolled were eligible for inclusion. Two reviewers (SY and MF-D) independently reviewed articles for inclusion.

Ethical approval was obtained from all participating sites for all studies included in the IPD analysis, and patients or their legal representatives provided written, informed consent according to national and local regulations. Ethical approval to perform the present IPD analysis was obtained from the site of the corresponding author. All data in the dataset are de-identified.

The primary efficacy outcome was moderately severe or severe disability or death at 90 days as defined by an mRS score of 4–6, hereafter referred to as “death or disability”. Only patients with the prestroke mRS score <4 were included for this analysis. The secondary efficacy outcome was hematoma expansion, defined as an increase in volume >6 cm^3^ from baseline to the 24 h CT scan [7]. Safety outcomes were any serious adverse events (SAEs) within 90 days and SAEs related to cardiac and renal dysfunction within 72 h.

### Statistical analysis

The mean level of hourly SBP between 1 h and 24 h after entry/randomization was determined in each patient. When SBP was measured more than once in an hour, the average value was taken as the hourly level. Patients with SBP data available for <4 timepoints were excluded from the analysis.

As an IPD meta-analysis to determine any significant independent associations of mean hourly SBP levels or their quartile levels with outcomes, the logistic regression model was adjusted for the variables used in our previous studies; [6] for sex, age, and study groups (model 1) or further for baseline SBP, baseline National Institutes of Health Stroke Scale (NIHSS) score (ranging from 0 to 42 with higher scores indicating worse neurological deficits), baseline hematoma volume, hematoma site (lobar or not), and onset-to-randomization time (<180 min or ≥180 min, model 2). Patients with unavailable data for the 90-day mRS score and those undergoing surgical procedures before the 24 h CT were excluded from the initial analysis of the primary and secondary efficacy outcomes, respectively. As the additional analyses, the former patients were regarded as having mRS scores of 4–6 and the latter were regarded as having hematoma expansion and included in the analyses. Effect modification by predefined subgroups on the association between mean hourly SBP and outcomes was identified by logistic regression using the multiplicative interaction terms (SBP × subgroups).

A value of *p* < 0.15 was considered significant for assessment of the treatment-by-subgroup interaction. In the other comparisons, a value of *p* < 0.05 was considered significant. Statistical analysis was performed using JMP version 16 software (SAS Institute).

## Results

**Fig. 1:**
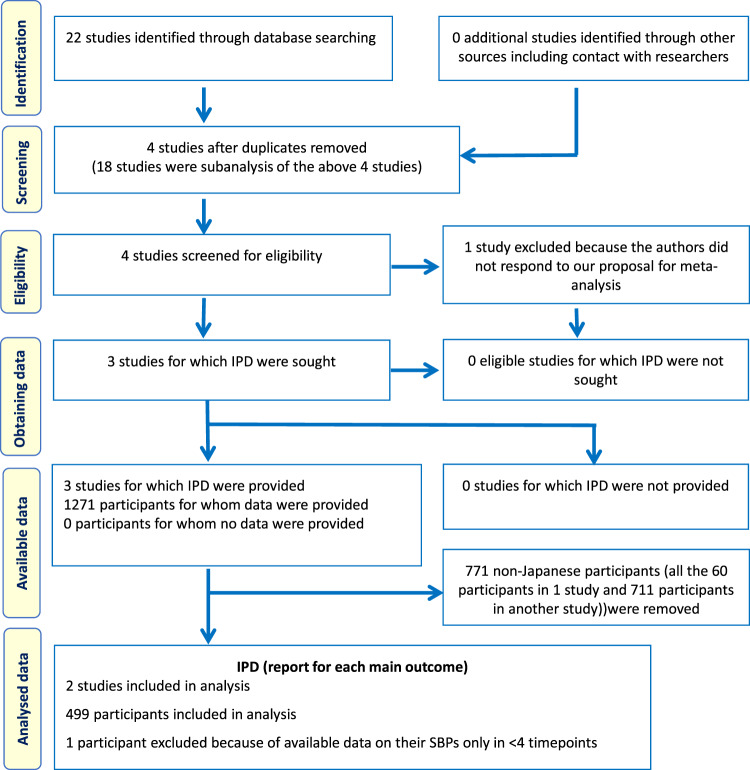
PRISMA individual participant data flow diagram

Three studies met the eligibility criteria: Antihypertensive Treatment of Acute Cerebral Hemorrhage (ATACH)-1 [8, 9], ATACH-2 [2, 10], and Stroke Acute Management with Urgent Risk-factor Assessment and Improvement-IntraCerebral Hemorrhage (SAMURAI-ICH, Fig. 1) [11]. Since ATACH-1 did not include Japanese patients, the present analysis was performed using the other two. Both studies enrolled adult patients with hyperacute spontaneous supratentorial ICH whose initial parenchymal hematoma volume was <60 mL, initial SBP was >180 mmHg, and initial Glasgow Coma Scale score was ≥5 and treated them with intravenous nicardipine during the initial 24 h using the titration method common to both studies (Supplementary Table 1). SBP was measured at least every 15 min during the first hour and every 15–30 min in ATACH-2 and at least every 60 min in SAMURAI-ICH during the remainder of the first 24 h. Major differences in methods were maximal allowed time from symptom onset to initiation of nicardipine treatment (4.5 h in ATACH-2 and 3 h in SAMURAI-ICH) and target SBP range during the first 24 h (two arms of 140–179 and 110–139 mmHg in ATACH-2 and a single arm of 120–160 mmHg in SAMURAI-ICH).

All 211 patients enrolled in SAMURAI-ICH and 289 of 1000 patients enrolled in ATACH-2 were Japanese. Of these, one patient with available data on their hourly SBPs only for <4 timepoints from ATACH-2 was excluded. Thus, 499 patients (183 women, 64.9 ± 11.8 years of age) were finally analyzed. The baseline characteristics of the patients are shown in Table 1. The trend of hourly SBP is shown in Supplementary Fig. 1. Mean baseline systolic/diastolic BP was 203.5 ± 18.3/109.1 ± 17.2 mmHg. Median hourly SBP between 1 h and 24 h was 134.3 mmHg (interquartile range 127.5–145.1 mmHg); these three values were the cutoffs of the SBP quartiles.

**Table 1:** Patients’ baseline characteristics

	*N* = 499
Women	183 (36.7%)
Age, y	64.9 ± 11.8
Asian race	499 (100%)
History of stroke/transient ischemic attack	66 (13.2%)
Hypertension	403 (80.9%)
Dyslipidaemia	125 (25.2%)
Diabetes mellitus	70 (14.1%)
Current smoking	153 (30.7%)
Glasgow Coma Scale score	15 [13–15]
National Institutes of Health Stroke Scale score	11 [7–16]
Hematoma volume, mL	9.4 [5.0–18.1]
Hematoma location	
Lobar	32 (6.4%)
Basal ganglia^a^	271 (54.3%)
Thalamus	197 (39.4%)
Intraventricular hemorrhage	104 (20.8%)
Onset-to-randomization, min	120 [90–165]

*n* (%), mean ± standard deviation or median [interquartile range]

^a^Mainly putaminal hematoma. Patients with mix hematoma in both putamen and thalamus is counted here. A patient with multiple hematomas in putamen, thalamus, and lobe is counted twice in “Lobar” and “Basal ganglia”

**Fig. 2:**
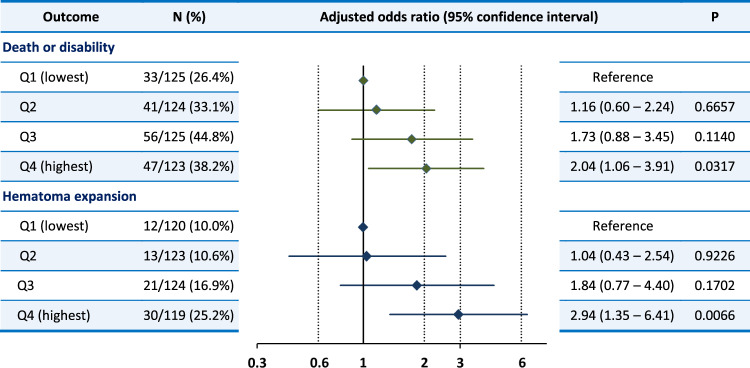
The association of mean hourly systolic blood pressure quartiles with outcomes Systolic blood pressure range: Q1, <127.5 mmHg; Q2, 127.5–134.3 mmHg; Q3, 134.3–145.1 mmHg; Q4, ≥145.1 mmHg. Adjusted for sex, age, study group, baseline systolic blood pressure, baseline National Institutes of Health Stroke Scale score, baseline hematoma volume, lobar hematoma, and onset-to-randomization time (model 2)

**Fig. 3:**
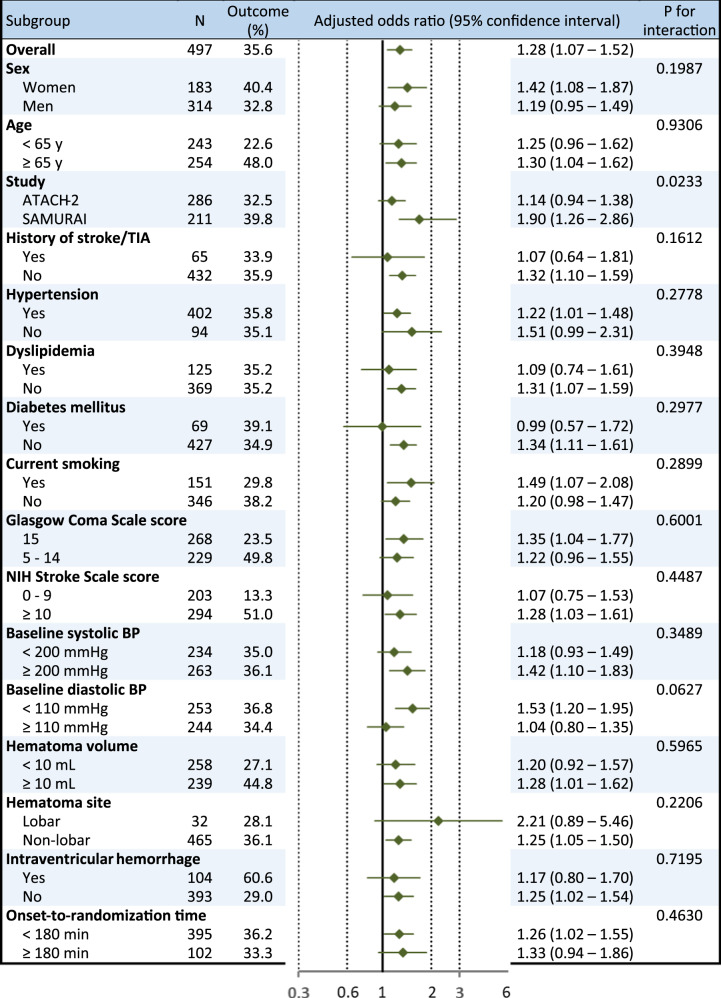
Associations of mean hourly systolic blood pressure with death or disability by subgroups. Adjusted for sex, age, and study groups

At 90 days, the mRS score was 0 in 30 patients, 1 in 93, 2 in 98, 3 in 99, 4 in 142, 5 in 29, 6 (death) in 7, and unavailable in 2. The proportion for the primary efficacy outcome of death or disability was 35.6% after exclusion of 2 patients with prestroke mRS of 4 (Table 2). After multivariable adjustment, a higher mean hourly SBP significantly increased the risk of death or disability [adjusted odds ratio (aOR) 1.25, 95% confidence interval (CI) 1.03–1.52 per 10 mmHg in model 2, Table 2]. The result was almost identical when two patients with unavailable data for the mRS score were regarded as having mRS scores of 4–6 and included. In the analysis using the quartiles of mean hourly SBP, the risk of death or disability gradually increased as SBP levels increased with the significant difference between the highest and lowest quartiles (aOR 2.04, 95% CI 1.06–3.91, Fig. 2). Study groups and diastolic BP modified the association between mean hourly SBP and the outcome (Fig. 3). Higher mean hourly SBP significantly increased the risk of death or disability in SAMURAI-ICH participants (aOR 1.90, 95% CI 1.26–2.86) and patients with diastolic BP < 110 mmHg (1.53, 1.20–1.95).

**Table 2:** The association of mean hourly systolic blood pressure between 1 and 24 h with outcomes

Outcome	*N* (%)	Crude			Adjusted. Model 1			Adjusted. Model 2		
		Odds ratio^a^	95% CI^a^	*P*	Odds ratio^a^	95% CI^a^	*P*	Odds ratio^a^	95% CI^a^	*P*
Death or disability	176/495 (35.6%)	1.17	1.00–1.37	0.0507	1.28	1.07–1.52	0.0051	1.25	1.03–1.52	0.0224
Adding 2 patients without available data^b^	178/497 (35.8%)	1.17	1.00–1.36	0.0538	1.28	1.08–1.52	0.0050	1.25	1.03–1.52	0.0221
Hematoma expansion	76/486 (15.6%)	1.51	1.22–1.86	0.0001	1.45	1.16–1.81	0.0011	1.49	1.18–1.87	0.0005
Adding 13 patients with emergent surgery^c^	89/499 (17.8%)	1.44	1.18–1.75	0.0003	1.38	1.13–1.68	0.0015	1.40	1.14–1.73	0.0014
Serious adverse events	88/499 (17.6%)	1.07	0.88–1.30	0.5114	1.11	0.90–1.36	0.3418	1.08	0.87–1.34	0.4892
Cardio-renal serious adverse events	7/499 (1.4%)	1.02	0.54–1.92	0.9478		—			—	

Multivariable analysis is not done for cardio-renal serious adverse events because of a small event number

Model 1: adjusted for sex, age, and study group

Model 2: adjusted for sex, age, study group, baseline systolic blood pressure, baseline National Institutes of Health Stroke Scale score, baseline hematoma volume, lobar hematoma, and onset-to-randomization time

^a^per 10 mmHg

^b^2 patients added are regarded as having death or disability

^c^13 patients added are regarded as having hematoma expansion

**Fig. 4:**
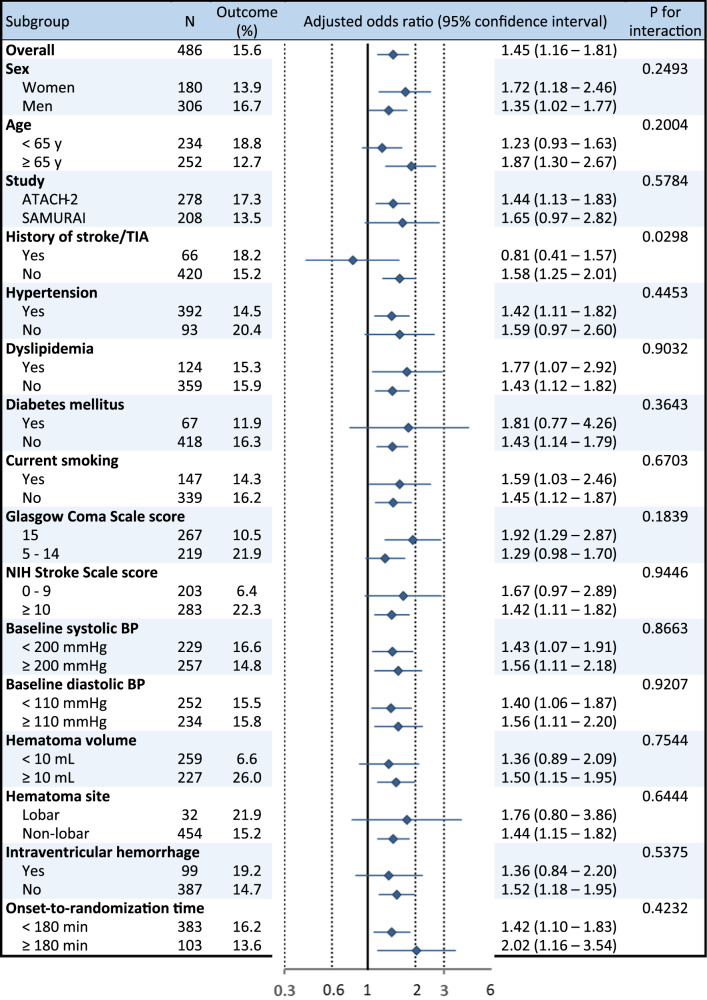
Associations of mean hourly systolic blood pressure with hematoma expansion by subgroups. Adjusted for sex, age, and study groups

The secondary efficacy outcome of hematoma expansion was not assessed in 13 patients due to emergent surgery within 24 h that might affect hematoma size. The proportion of hematoma expansion in the other 486 patients was 15.6% (Table 2). Mean hourly SBP was positively and significantly associated with the risk of hematoma expansion (aOR 1.49, 95% CI 1.18–1.87 per 10 mmHg in model 2). The result was similar when all 13 patients with emergent surgery were regarded as having hematoma expansion (1.40, 1.14–1.73). In the analysis using the quartiles of mean hourly SBP, the risk of hematoma expansion gradually increased as SBP levels increased with the significant difference between the highest and lowest quartiles (aOR 2.94, 95% CI 1.35–6.41, Fig. 2). History of stroke/transient ischemic attack showed significant treatment-by-subgroup interactions in relation to hematoma expansion (Fig. 4).

SAEs occurred in 17.6% within 90 days and cardio-renal SAEs in 1.4% within 72 h among all 499 patients (Table 2). Mean hourly SBP was not significantly associated with either any SAEs or cardio-renal SAEs.

## Discussion

In the present IPD meta-analysis, the association of acute SBP levels achieved with continuous intravenous administration of nicardipine for Japanese ICH patients on clinical outcomes were explored. The major finding was that a lower level of mean hourly SBP achieved during the initial 24 h by nicardipine was associated with lower risks of hematoma expansion defined as an increase in volume >6 cm^3^ on the 24 h CT scan and moderately severe or severe disability or death at 90 days as defined by an mRS score of 4–6. The similar findings were obtained for both of these two outcomes in the analysis for the categorized mean hourly SBP level. The risk of SAEs did not change according to achieved SBP. The finding had been also proven in overall global ICH patients registered in the same IPD meta-analysis [6]. Interestingly, the impact of SBP lowering on better outcome seemed to be stronger in Japanese patients than the global ones.

Two large, global, randomized, controlled trials, the Second Intensive Blood Pressure Reduction in Acute Cerebral Haemorrhage Trial (INTERACT2) and the ATACH-2 trial, were published in succession in the mid-2010’s [1, 2]. INTERACT2 demonstrated possibly better functional outcomes for acute ICH patients with early intensive lowering to a target SBP of <140 mmHg than the standard SBP target of <180 mmHg (odds ratio 0.87, 95% CI 0.75–1.01) [1]. In contrast, ATACH-2 did not show benefit in reducing the rate of death or disability between the two treatment groups with the same target SBP level as INTERACT2 (relative risk with intensive treatment 1.04, 95% CI 0.85–1.27) [2]. In a pooled analysis of these two trials, the level of SBP in the initial 24 h was continuously associated with the distribution of the mRS scores at 90 days [12]. SAMURAI-ICH was a kind of pilot study of ATACH-2 that aimed to prove the safety of nicardipine use in Japanese ICH patients in the period when intravenous nicardipine for hyperacute ICH patients was limited on the official label in Japan without scientific evidence [3, 11]. Study designs of SAMURAI-ICH were accordingly similar with that of ATACH-2.

Hematoma expansion was reported to greatly attenuate by deep and rapid SBP lowering [12–15]. The positive and significant association between mean hourly SBP and the risk of hematoma expansion was also evident in the overall participants of the present IPD meta-analysis mainly from the United States and East Asia [6]. The impact of the association was somewhat different; the aOR was 1.16 (95% CI 1.02–1.32 per 10 mmHg) in the overall result and 1.49 (1.18–1.87) in the present one limited to Japanese patients. As compared to the overall participants, Japanese had relatively short time delay from onset to randomization (median 120 min versus 168 min), suggesting the higher rate of active continuous bleeding at randomization in the Japanese cohort [16–18]. For such patients with short time delay, intensive SBP lowering may be much protective against hematoma expansion. In the analysis of SAMURAI-ICH alone, the lowest quartile of time from imaging to target SBP < 160 mmHg (<38 min) was negatively associated with hematoma expansion [19]. When the present patients <120 min time delay were separately analyzed, the aOR for the association of SBP with hematoma expansion was 1.60 (95% CI 1.09–2.36). In addition, etiological mechanism of spontaneous ICH is considered to be more hypertensive and less amyloid-angiopathic in Japanese than western population [20]. This may be another reason for strong association of SBP level and hematoma expansion.

The impact of the association between mean hourly SBP and the risk of death or disability at 90 days was also somewhat stronger in the present Japanese patients (aOR 1.25, 95% CI 1.03–1.52) relative to the overall participants of the IPD meta-analysis (1.12, 1.00–1.26) [6]. It seems to reflect the known finding that hematoma expansion is strongly related to poor functional outcomes [21]. When the present patients with <120 min time delay were separately analyzed, the aOR for the association of SBP with death or disability was 1.63 (95% CI 1.19–2.21) and showed a significant treatment-by-subgroup interaction as compared to those ≥120 min delay (aOR 1.14, 95% CI 0.93–1.41, P for interaction = 0.1211).

Subgroup analyses shown in Figs. 3 and 4 did not show the similar tendency. Although history of stroke/transient ischemic attack modified the association of mean hourly SBP with hematoma expansion, it did not modify those with death or disability. A small number of patients with the history might cause the statistical bias. Subgroups divided by baseline diastolic BP level (<110 mmHg versus ≥110 mmHg) had the similar association of SBP with hematoma expansion, but showed a significant treatment-by-subgroup interaction in relation to death or disability. The similar finding was shown in the global participants of the IPD meta-analysis [6].

The limitations of the IPD meta-analysis used here were described elsewhere, including the difficulty in generalization of results to very severe ICH patients because of exclusion of patients with supratentorial hemorrhage ≥60 mL in volume and those with infratentorial hemorrhage from the study [6]. In addition, merging the datasets of the randomized ATACH-2 and non-randomized SAMURAI-ICH together might cause potential bias. In the present subgroup analysis, the association of SBP with death or disability differed between ATACH-2 and SAMURAI-ICH (P for interaction = 0.0233), although the association of SBP with hematoma expansion was not so different between the two studies.

In conclusion, the present Japanese patients with acute ICH extracted from the database of our IPD meta-analysis showed somewhat stronger association of the lower mean SBP level within the initial 24 h with better imaging and functional outcomes as compared to the global cohort from the same meta-analysis. Acute SBP lowering was not associated with the increase in SAEs. However, we should still take care of the previous lesson from ATACH-2 that intensive SBP lowering is potentially harmful among patients with a decreased estimated glomerular filtration rate, that are relatively common in Japanese [22].

## Supplementary information

Supplementary Table and Figure
