# Supplementary material for: Intravenous nicardipine for Japanese patients with acute intracerebral hemorrhage: an individual participant data analysis
Source: Hypertens Res. 2022 Oct 13;46(1):75–83. doi: 10.1038/s41440-022-01046-4 (PMC9747609; doi:10.1038/s41440-022-01046-4)
Supplement: Supplementary file 1 — Supplementary Table and Figure [file 41440_2022_1046_MOESM1_ESM.pdf]

Supplementary Table 1. Design and main methods of the individual studies

	ATACH-2	SAMURAI-ICH
Design	Randomized, two-group, open-label	One-arm, prospective
Registration period	May 2011 – September 2015	July 2009 – June 2011
Number of registered patients	1,000	211
Target disease	Intracerebral hemorrhage, spontaneous, supratentorial, hematoma volume <60 mL	
Age / sex	≥18 y, <90 y, either sex	≥20 y, either sex
Initial systolic blood pressure (SBP)	>180 mmHg	
Initial Glasgow Coma Scale	≥5	
Inclusion of patients taking warfarin	Eligible unless taking within the last 5 d	Eligible if INR is <1.7
Treatment	Continuous intravenous nicardipine for 24 h	
Max time from onset to nicardipine	4.5 h (initially 3 h)	3 h
Target range of SBP	[1] 140 – 179 mmHg versus [2] 110 – 139 mmHg (1:1)	120 – 160 mmHg
Titration of nicardipine	Nicardipine was administered as a continuous infusion with a starting dose of 5 mg/h, and then increased by 2.5 mg/h every 15 min as needed, up to a maximum of 15 mg/h. If SBP fell below the lower limit of target range, nicardipine was reduced in a stepwise pattern until SBP returns to target range or nicardipine is discontinued.	
Frequency of SBP measurement	Every 5 min during the first 15 mins, then every 15 min for the first hour, and every 15 to 30 min during the remainder of the first 24 h	Every 15 min during the first 2 h, and every 60 min during the remainder of the first 24 h
Timing of brain CT examination	Baseline and 24 h later	
Follow-up mRS	90 d (3 months)	

Supplementary Figure 1. The trend of hourly systolic blood pressure during the initial 24 h

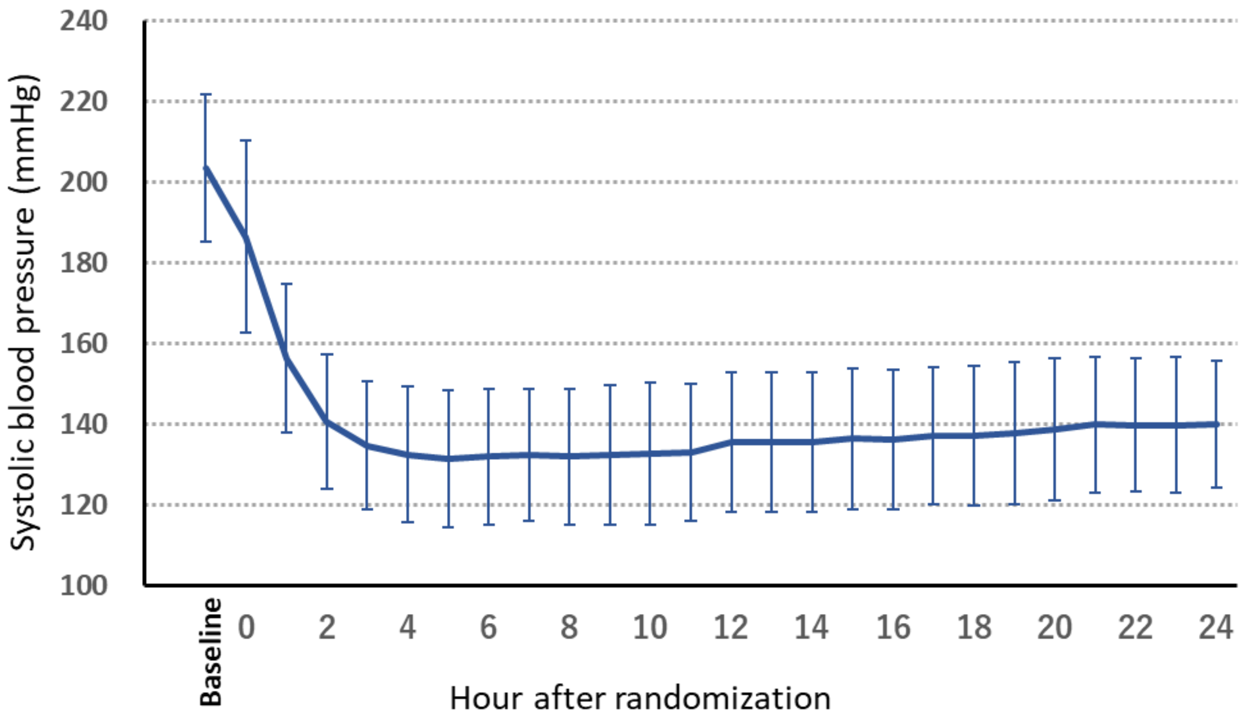

Mean values and 95% confidence intervals.
